# Improved strategy for post-traumatic hydrocephalus following decompressive craniectomy: Experience of a single center

**DOI:** 10.3389/fsurg.2022.935171

**Published:** 2023-01-06

**Authors:** Kun Wang, Hongbin Guo, Yinxin Zhu, Jinjian Li, Huanjiang Niu, Yirong Wang, Xiujun Cai

**Affiliations:** ^1^ Department of Neurosurgery, Sir Run Run Shaw Hospital, Medical College, Zhejiang University, Hangzhou, China; ^2^ Department of Neurosurgery, Hangzhou Xiasha Hospital, Sir Run Run Shaw Hospital, Medical College, Zhejiang University, Hangzhou, China; ^3^ Department of General Surgery, Sir Run Run Shaw Hospital, Medical College, Zhejiang University, Hangzhou, China

**Keywords:** post-traumatic hydrocephalus, cranioplasty, ventriculoperitoneal shunt, complication, procedure

## Abstract

**Background:**

Patients with head trauma may develop hydrocephalus after decompressive craniectomy. Many studies have referred one-stage cranioplasty (CP) and ventriculoperitoneal shunt (VPS) was applied to treat cranial defect with post-traumatic hydrocephalus (PTH), but the safety and efficiency of the procedure remain controversial.

**Methods:**

This is a retrospective cohort study including 70 patients of PTH following decompressive craniectomy who underwent simultaneous (50) and separated (20) procedures of cranioplasty and VPS from March 2014 to March 2021 at the authors’ institution with at least 30 days of follow-up. Patient characteristics, clinical findings, and complications were collected and analyzed.

**Results:**

Fifty patients with PTH underwent improved simultaneous procedures and 20 patients underwent staged surgeries. Among the cases, the overall complication rate was 22.86%. Complications suffered by patients who underwent one-stage procedure of CP and VPS did not differ significantly, compared with patients in the group of staged procedures (22% vs. 25%, *p* = 0.763). The significant difference was not observed in the two groups, regarding the complications of subdural/epidural fluid collection (4%/6% vs. 0/2%, *p* = 1.000/1.000), epidural hemorrhage (6% vs. 4%, *p* = 0.942), dysfunction of shunting system (0 vs. 2%, *p* = 0.286), postoperative seizure (8% vs. 4%, *p* = 1.000), and reoperation case (0 vs. 2%, *p* = 0.286). No case of subdural hemorrhage, incision/intracranial/abdominal infection, shunting system dysfunction, or reoperation was observed in the group of simultaneous procedure. Complications including subdural/epidural fluid collection, subdural hemorrhage, and incision/intracranial infection were not shown in the case series of the staged procedure group.

**Conclusion:**

The improved simultaneous procedure of cranioplasty and VPS is effective and safe to treat cranial defect and post-traumatic hydrocephalus with low risk of complications.

## Introduction

In China, 1.4 billion patients with traumatic brain injury (TBI) are cause of an enormous burden to society and families (1). In a global study, it was found that moderate and severe TBI patients accounted for 60% of all TBI patients, and the total mortality rate is 18%. According to the human development index (HDI), there is a certain correlation between mortality and HDI (2). Decompressive craniectomy (DC) is considered a typically used method to resolve medically refractory intracranial hypertension in patients suffering from head injury (3, 4). DC can decrease intracranial pressure, and thus improve cerebral perfusion pressure and oxygen supply (5–8). In addition, a large cranial defect could further lead to disturbance in cerebrospinal fluid (CSF) hydrodynamics and cerebral perfusion due to exposure to atmospheric pressure (9).

Post-traumatic hydrocephalus (PTH) is a devastating complication following DC, typically characterized by excessive accumulation of CSF and leading to disorders of CSF circulation. PTH may affect the function or metabolism of the central nervous system, clinical improvement, and outcomes of the patients, which necessitate the placement of a ventriculoperitoneal shunt (VPS) to relieve hydrocephalus (10). Therefore, it is valuable for the early diagnosis and treatment of PTH, which can reduce further neurological complication incidence in these patients.

VPS and cranioplasty (CP) are effective methods for PTH secondary to DC. However, there is still controversy on the ideal method for addressing both hydrocephalus and cranial defects. According to some reviews, one-stage procedure of VPS and cranioplasty was associated with a higher rate of complications compared with separate surgeries (11). In this study, we reported the clinical data and details of one-stage CP and VPS with minimization of complications applied in patients with cranial defects and hydrocephalus in our center.

## Methods

### Ethics approval and consent to participate

This study was performed in the Sir Run Run Shaw Hospital, Medical College, Zhejiang University, and was approved by the institutional review board. A retrospective review was conducted in 70 patients who underwent simultaneous cranioplasty and CSF shunt implantation within 6 months after a decompressive craniectomy for head trauma from March 2014 to March 2021. Baseline patient demographics, clinical characteristics, and complication rates are shown in Tables 1, 2. All the patients or their family provided informed consent, which contained a detailed description of the purpose of this study. All the information of the cases were handled and made anonymous according to ethical and legal standards.

**Table 1:** Patient demographics and clinical characteristics.

Characteristics	Simultaneous procedure	Staged procedure	*P* value
	Group (*n* = 50)	Group (*n* = 20)	
No. patients			
Male	35	11	0.272
Female	15	9	
Mean age (years)	54.98 ± 14.95	59.30 ± 14.56	0.712
Mean interval between DC and subsequent op (months)	2.50 ± 0.85	2.78 ± 1.15	0.435
Cranial defect size			
Smaller than half of hemisphere	7	4	0.717
Larger than half of hemisphere	43	16	
Severity of hydrocephalus			
Bifrontal index < 0.4	28	8	0.293
Bifrontal index > 0.4	22	12	

DC, decompressive craniectomy.

**Table 2:** Comparison of secondary complications between two groups.

Complications	Simultaneous procedure	Staged procedure	*p* value
	Group (*n* = 50)	Group (*n* = 20)	
Incidence of complications	22% (11/50)	25% (5/20)	0.763
Subdural/epidural fluid collection	2/3	0/1	1.000/1.000
Subdural/epidural hemorrhage	0/3	0/2	−/0.942
Incision/intracranial/infection	0	0	—
Shunting system dysfunction	0	1	0.286
Postoperative seizure	4	2	1.000
Reoperation case	0	1	0.286

### Evaluation of post-traumatic hydrocephalus

Hydrocephalus developing within 6 months following decompressive craniectomy was defined as PTH, excluding enlargement of the ventricular system not attributable to brain atrophy (12). The diagnostic criteria are as follows: (1) Evans index is the ratio of the ventricular width of the bilateral frontal horn to the maximum biparietal diameter, which is greater than 0.3 on the radiological examinations; (2) progressive enlargement of ventricular system; (3) periventricular decreased density on head CT scan; (4) neurological improvement after lumbar puncture for the withdrawal of cerebrospinal fluid. The severity of PTH was evaluated *via* the Evans index. An index greater than 0.4 was regarded as severe hydrocephalus.

### Improved surgical strategies for the cranial defect and post-traumatic hydrocephalus

**Figure 1:**
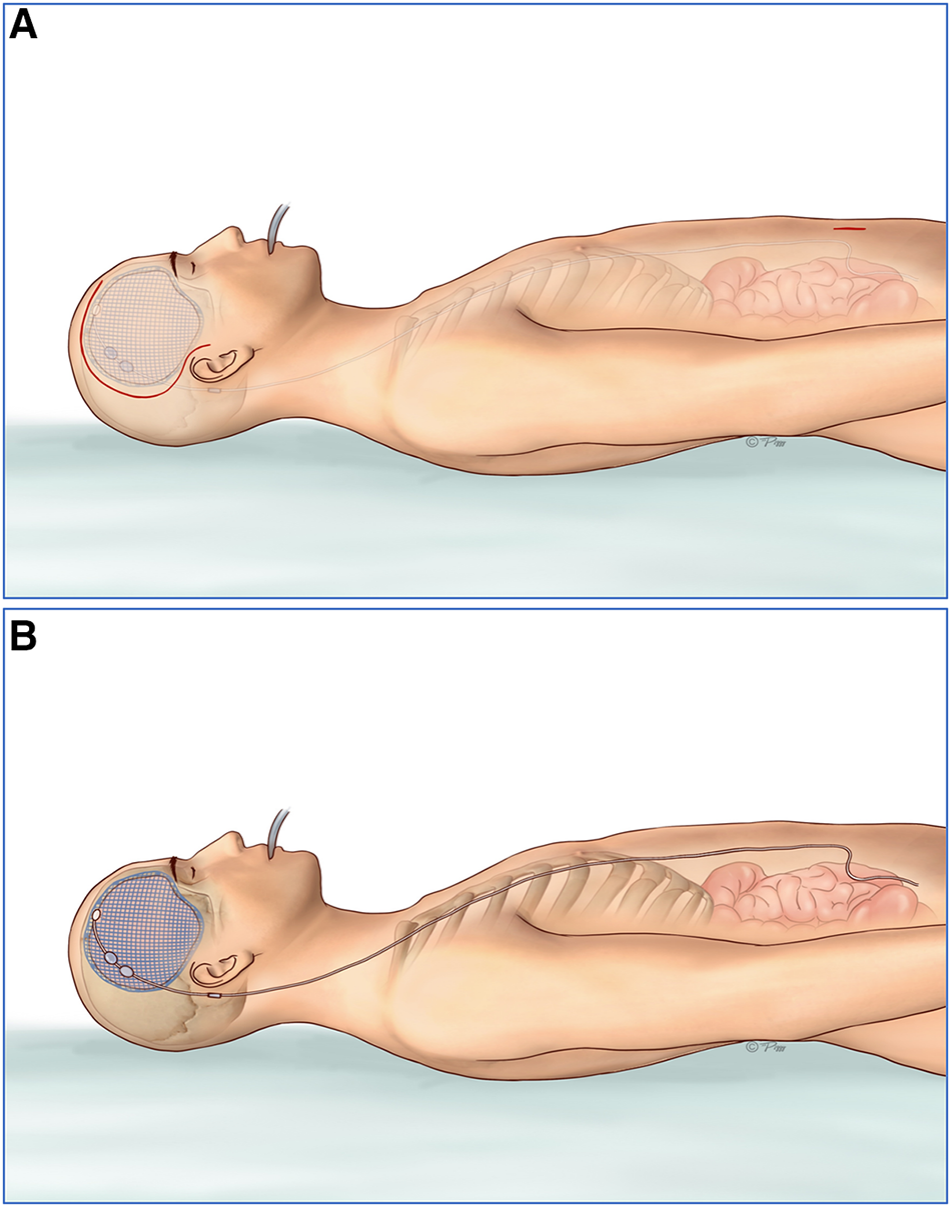
(**A**) Two incisions were made in the frontotemporal and paraumbilical region. (**B**) Three-dimensional plastic titanium mesh was used in the operation as cranial repair material. Programmable ventriculoperitoneal shunt of Aesculap was guided by the Medtronic surgical neuronavigation system and performed in the same side of the cranioplasty. The connection of the reservoir, adjustable unit, gravitational unit, and tube was improved to ensure a better location of the shunting parts, gravitational unit vertical to the ground just behind the ear, smoothing angle of the tube, and minimalization of the surgical injury. DP, differential pressure.

Fifty patients with post-traumatic hydrocephalus following decompressive craniectomy underwent the improved one-stage procedure and 20 patients underwent separate surgeries. The same surgical technique with the shunt on the same side of the cranioplasty was applied in the patients of the simultaneous group, while the shunt was placed contralaterally in the staged groups. Two incisions were made in the frontotemporal and paraumbilical region. A three-dimensional plastic titanium mesh (13) was used in the operation as cranial repair material. Implantation of programmable ventriculoperitoneal shunt (Aesculap) was guided by the Medtronic surgical neuronavigation system and performed in the same side of the cranioplasty (Figure 1). The connection of the reservoir, adjustable differential pressure (DP) unit, gravitational unit, and tube was improved to ensure a better location of the shunting parts, gravitational unit vertical to the ground just behind the ear, smoothing angle of the tube, and minimalization of surgical injury (Figure 1). There were two reasons why we chose the same side for the VPS: (1) Injury could be decreased if VPS placement was in the same side with cranioplasty. The operation time and hospital stays would be shortened and the medical and social resources would be saved, compared with the staged procedure. (2) There would remain another contralateral site for another VPS placement if the first shunting system was dysfunctional. The pressure of a programmable shunt valve was initially set at 160 mm H_2_O prior to operation and was gradually lowered by reference to brain CT about 2 weeks after the surgery. Postsurgical head CT scan was commonly performed within 24 h.

**Figure 2:**
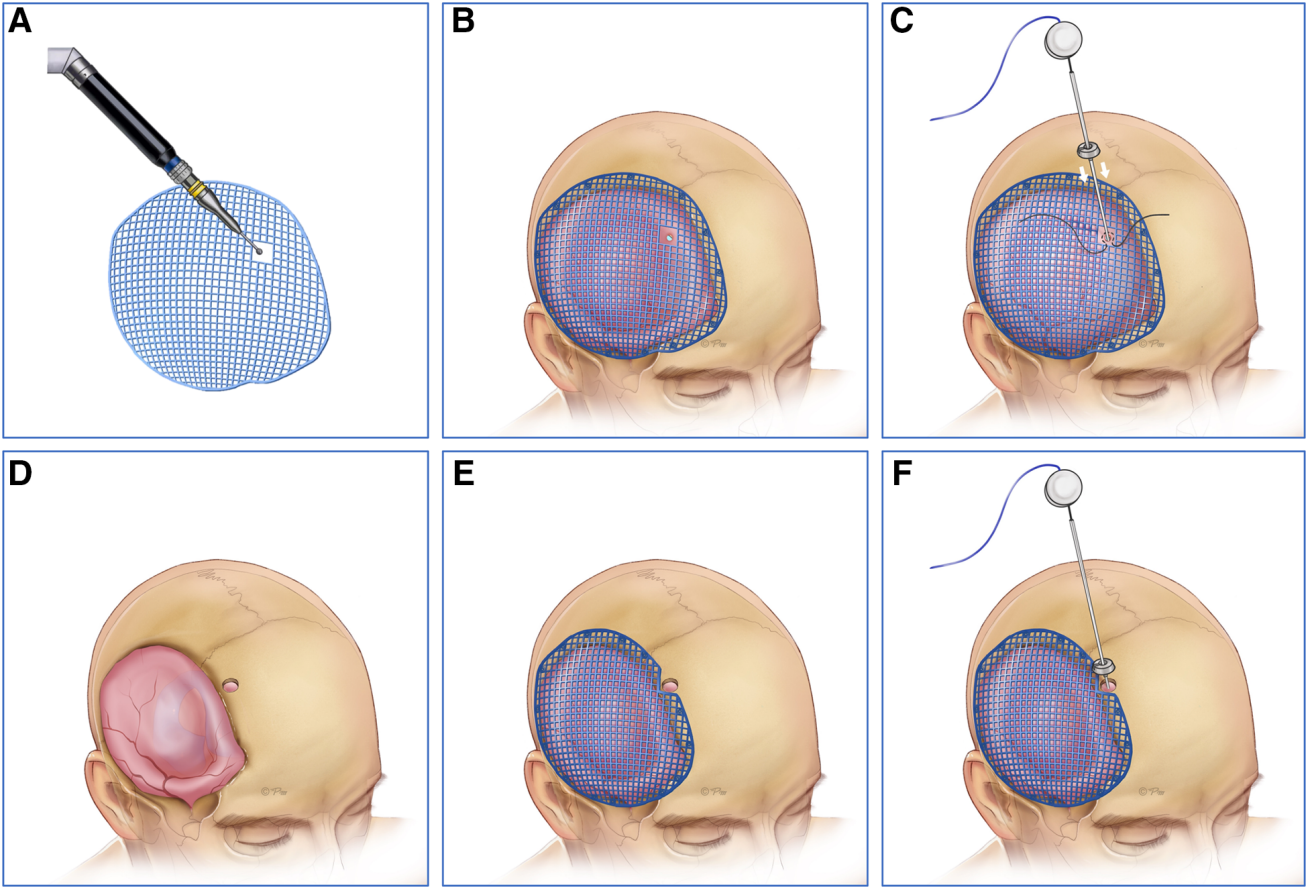
(**A**,**D**) The neuronavigation system was first used to identify the entry point in the titanium mesh or the frontal bone margin. (**B,E**) Then, we performed cranioplasty and suspended the titanium mesh and dura mater with silk thread to prevent the epidural hematoma/fluid collection. (**C,F**) Finally VP shunt implantation was performed assisted by the neuronavigation system. VP, ventriculoperitoneal.

1. Flaccid concave cranial defect and flaccid with partial convex cranial defect:

A flaccid concave cranial defect or flaccid with a partial convex cranial defect was defined if all the brain tissue was under the skull line or part of the brain was bulging above the skull line. In this situation, we would perform cranioplasty first, and then suspend the titanium mesh and dura mater with silk thread to prevent the epidural hematoma/fluid collection. A neuronavigation system was used to identify the entry point in the titanium mesh or the frontal bone margin. Then, the VP shunt implantation was performed assisted by the neuronavigation system (Figure 2).

**Figure 3:**
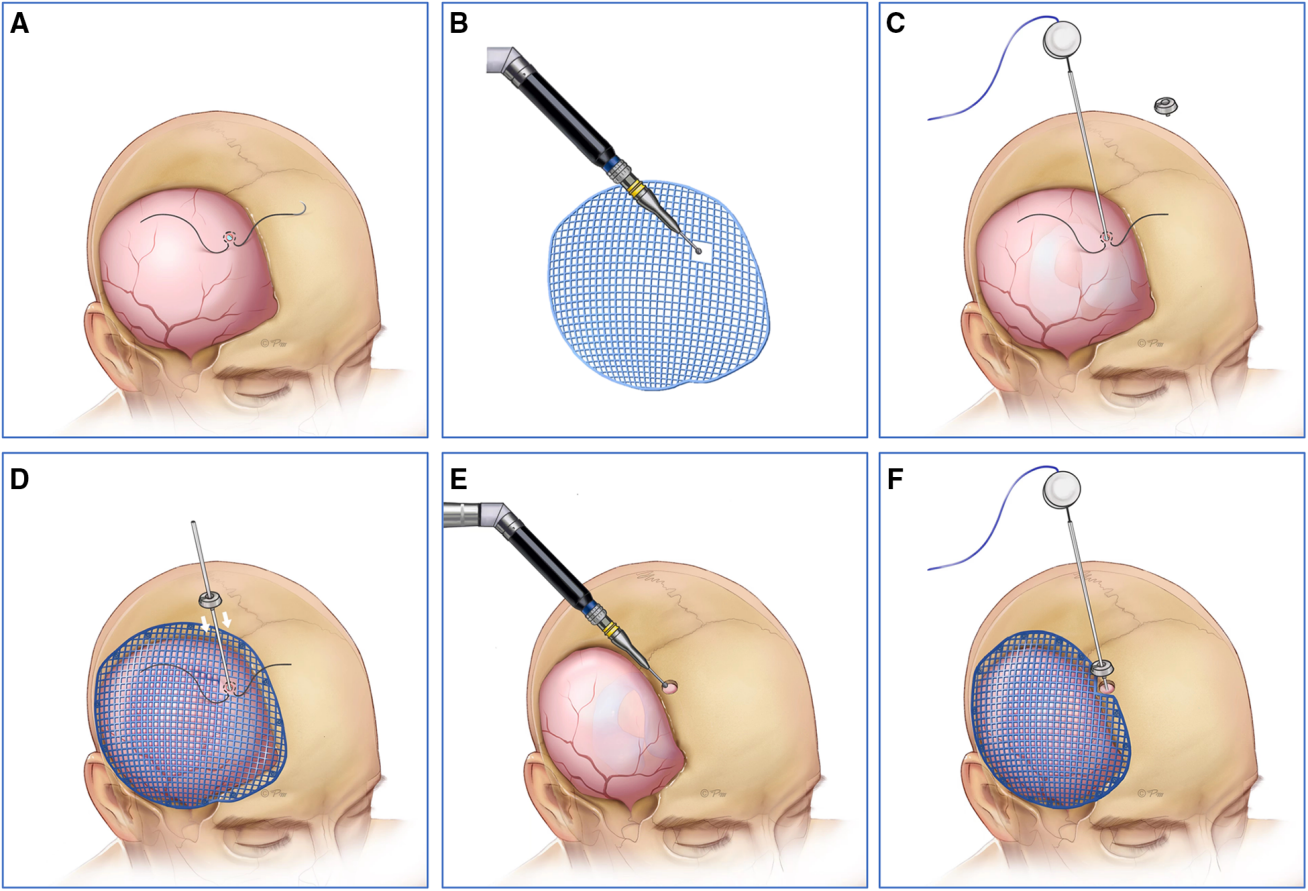
(**A,B,E**) First, the entry point was located by neuronavigation system in the dura matter or the frontal bone margin in the same side of cranioplasty. (**C,D,F**) Then, the shunting tube was inserted to release the cerebrospinal fluid and the intracranial pressure was decreased under neuronavigation system. Finally, the titanium mesh was attached to the dura matter perfectly, and the shunting device was connected.

2. Tense convex cranial defect:

A tense convex cranial defect was defined if all the brain tissue was above the skull line. In almost all of the cases, cranioplasty should not be the first choice because of severe brain bulging and high intracranial pressure. We solved the problem effectively by three steps in the one-stage surgery. First, the entry point was located by the neuronavigation system in the dura matter or the frontal bone margin in the same side of cranioplasty. Then, the shunting tube was inserted to release the cerebrospinal fluid and decrease the intracranial pressure. Finally, the titanium mesh was attached to the dura matter perfectly, and the shunting devices were connected (Figure 3).

### Evaluation of complications secondary to simultaneous and separated procedures

The complications secondary to one-stage and staged procedures included epidural/subdural fluid collection, epidural/subdural hematomas, incision/intracranial/abdominal infection, malfunction of the shunting device, postoperative seizure, and reoperation. If the cranial repair material or shunting device was removed, severe neurological impairment occurred, or even death was triggered because of surgical complications, the former operation was considered to a failure.

### Statistical method

Data analyses were performed using SPSS software (version 19, SPSS, Inc.). Continuous variables were compared using Student's *t* tests and chi-square test or Fisher's exact test for discrete variables. *p* values <0.05 were considered to indicate statistical significance.

## Results

### Clinical characteristics and secondary complications

In the case database of Sir Run Run Shaw Hospital affiliated to the Medical College of Zhejiang University from March 2014 to March 2021, 50 patients with PTH following DC underwent improved simultaneous procedures and 20 patients underwent staged surgeries. Results of the two groups did not show any significant difference regarding gender and age. The mean intervals between DC and subsequent operation in the two groups were 2.50 ± 0.85 and 2.78 ± 1.15 months (*p* > 0.05). The cranial defect size was assessed by “smaller than half of a hemisphere” and “larger than half of a hemisphere.” Statistical significance of the size of cranial defect was not revealed in the two groups (*p* > 0.05). The severity of hydrocephalus was evaluated by the Evans index (greater or smaller than 0.4), which did not show any difference between the two groups (Table 1).

Postoperative complications are shown in Table 2. Among the patients, the overall complication rate was 22.86%. Complications suffered by patients who underwent simultaneous procedure of CP and VPS did not differ significantly, compared with patients in the group of staged procedures (22% vs. 25%, *p* = 0.763). Complications occurred in 11 patients (22%, 11/50) in the simultaneous procedure group, including 2 cases of subdural fluid collection, 3 cases of epidural fluid collection, 3 cases of epidural hemorrhage, and 4 cases of postoperative seizure. All the patients received medical conservative treatment and were discharged from the hospital after showing improvement. No case was observed with regard to subdural hemorrhage, incision/intracranial/abdominal infection, shunting system dysfunction, and reoperation rate. In the staged procedure group, five patients (25%, 5/20) developed secondary complications, including one case of epidural fluid collection, two cases of epidural hemorrhage, one case of shunting system dysfunction, two cases of postoperative seizure, and one patient underwent reoperation because of shunting system dysfunction. The shunting device was removed in one patient with the implantation of VPS simultaneously. No patient suffered from subdural fluid collection, subdural hemorrhage, and incision/intracranial/abdominal infection. The failure rate was thus 5% (1/20) in the latter group. No difference was observed significantly in the two groups regarding the complications of subdural/epidural fluid collection (4%/6% vs. 0/2%, *p* = 1.000/1.000), epidural hemorrhage (6% vs. 4%, *p* = 0.942), dysfunction of shunting system (0 vs. 2%, *p* = 0.286), postoperative seizure (8% vs. 4%, *p* = 1.000), and reoperation (0 vs. 2%, *p* = 0.286).

### Illustrative cases of one-stage CP and VPS

#### Case 1

**Figure 4:**
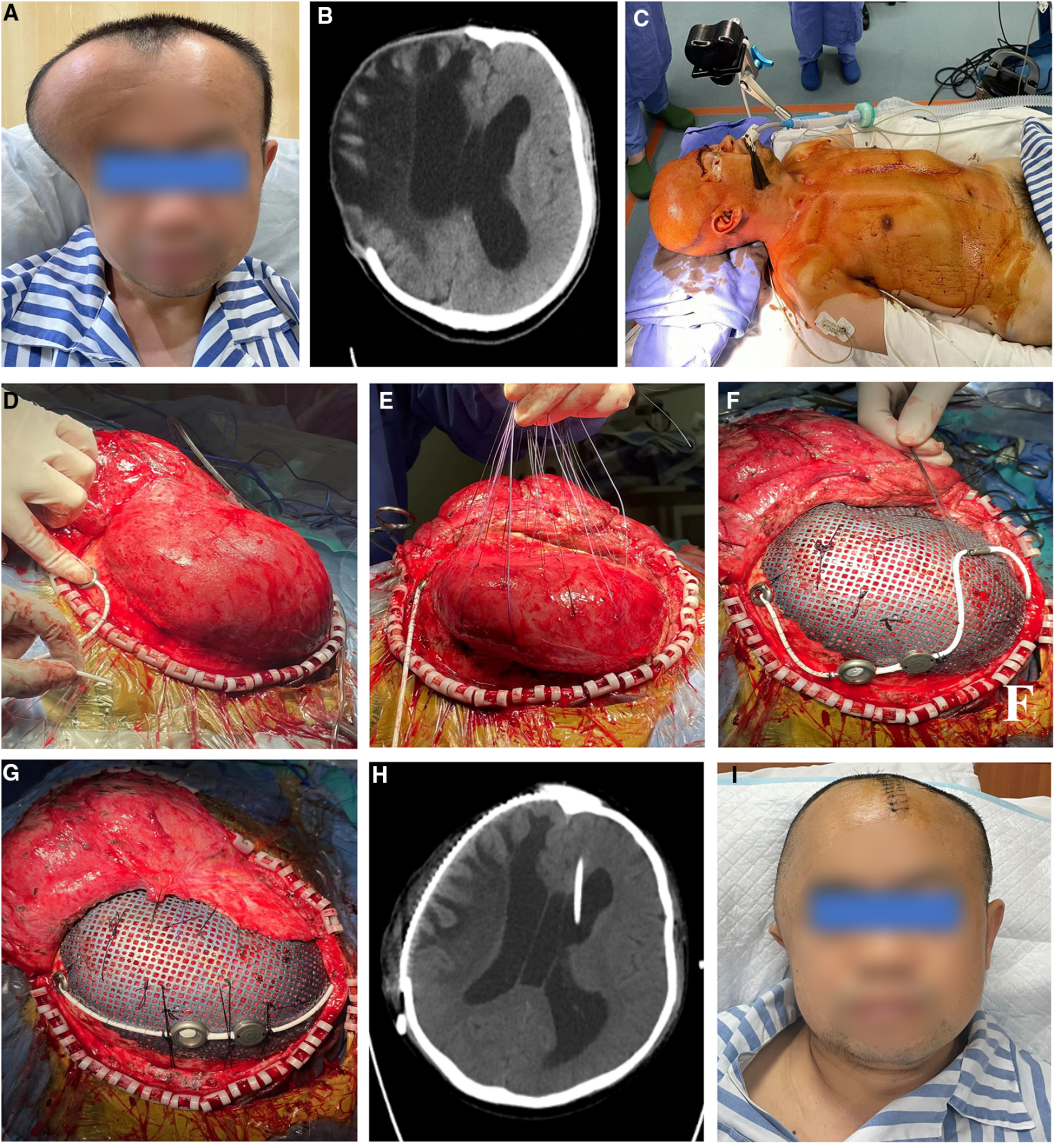
(**A**) severe encephalocele of the right frontotemporal region was observed. (**B**) Head CT scan revealed enlargement of the ventricular system. (**C**–**G**) Because of severe brain bulging, VP shunt was first implanted to release the CSF. The frontal ventricular puncture at the frontal bone was guided by the neuronavigation system. The parts of shunting devices were connected to the titanium mesh by an improved method. (**H**) Postoperative head CT showed no subdural/epidural fluid collection or subdural/epidural/cerebral hematoma. (**I**) The patient recovered rapidly and was discharged a week later. VP, ventriculoperitoneal; CSF, cerebrospinal fluid.

A 41-year-old male was admitted to the local hospital due to a head trauma. The head CT scan showed right-side acute subdural hematoma and cerebral contusion; further emergent craniectomy was performed to remove the hematoma and decrease the intracranial pressure (ICP). During the 3-month rehabilitation, serial CT scans revealed gradual enlargement of the ventricular system, which was diagnosed as hydrocephalus. The patient presented with headache, gait disturbance, and urinary incontinence. Severe encephalocele of the right frontotemporal region was observed (Figure 4A). The symptom improved significantly after cerebrospinal fluid tap test. Therefore, the patient was transferred to our department for subsequent evaluation and treatment. Radiological examination showed right skull defect and dilated ventricular system with periventricular edema (Figure 4B). The patient then underwent one-stage CP and VPS. The shunt pressure was presurgically set at 160 mm H_2_O to “dysfunction” the shunting system and avoid the overdrainage of CSF, which would lead to the formation of epidural hematoma between the titanium mesh and dura. Because of severe brain bulging, we performed VP shunting first to release the CSF. Frontal ventricular puncture to the frontal bone was guided by neuronavigation system (Figures 4C–E). The parts of the shunting device (reservoir, adjustable differential DP unit, gravitational unit, and tube) were connected on the titanium mesh by an improved method (Figure 4F). No subdural/epidural fluid collection or subdural/epidural/cerebral hematoma was observed from the postoperative head CT.

#### Case 2

**Figure 5:**
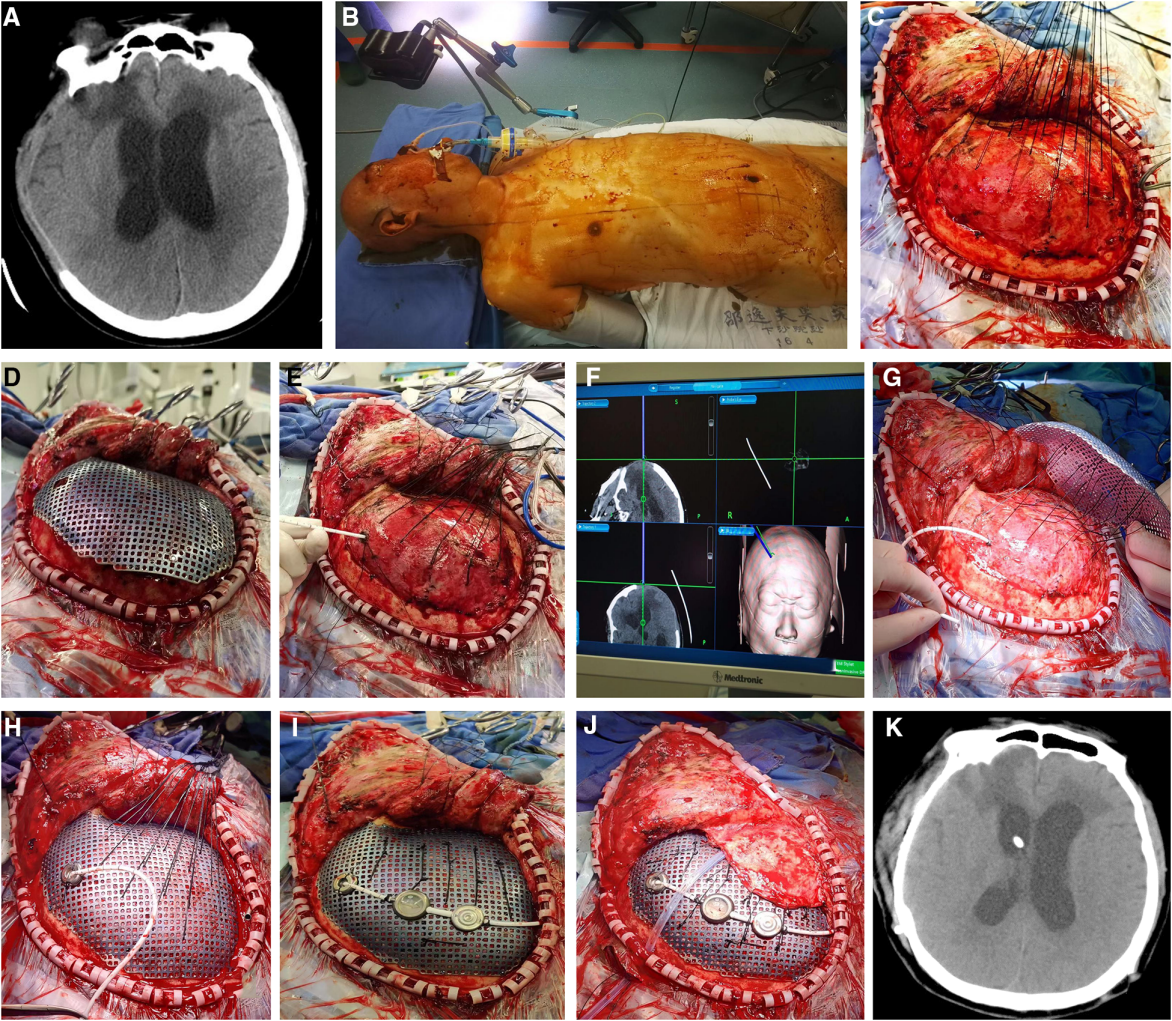
(**A**) Presurgical head CT scan demonstrated expansion of ventricular system, which was diagnosed as hydrocephalus. (**B–J**) Because of the encephalomeningocele, we first identified the entry point on the dura matter just close to the bone margin by the neuronavigation system. Then, ventricular puncture was performed to release the CSF till the titanium mesh could be attached to dura matter adequately, and parts of the shunting devices were further connected. (**K**) No operation-related complication was shown in the postsurgical radiological examinations. CSF, cerebrospinal fluid.

A 65-year-old male who suffered a head injury was transferred to our hospital. Head plain CT revealed right frontal and temporal cerebral contusion. Then, right craniectomy was performed to evacuate the cerebral contusion/hematoma. Two weeks later, the patient was discharged for rehabilitation therapy. During the period, serial CT scans revealed expansion of the ventricular system (Figure 5A). Because of the deteriorating level of consciousness, the patient came to our hospital 2 months later for treatment. Because of the encephalomeningocele, we first identified the entry point on the dura matter just close to the bone margin assisted by the neuronavigation system (Figures 5B,D). Then, ventricular puncture was performed to release the CSF till the titanium mesh could be attached to dura matter adequately, and parts of the shunting devices were connected (Figures 5E–J). No operation-related complication was observed in the postsurgical examinations (Figure 5K).

#### Case 3

**Figure 6:**
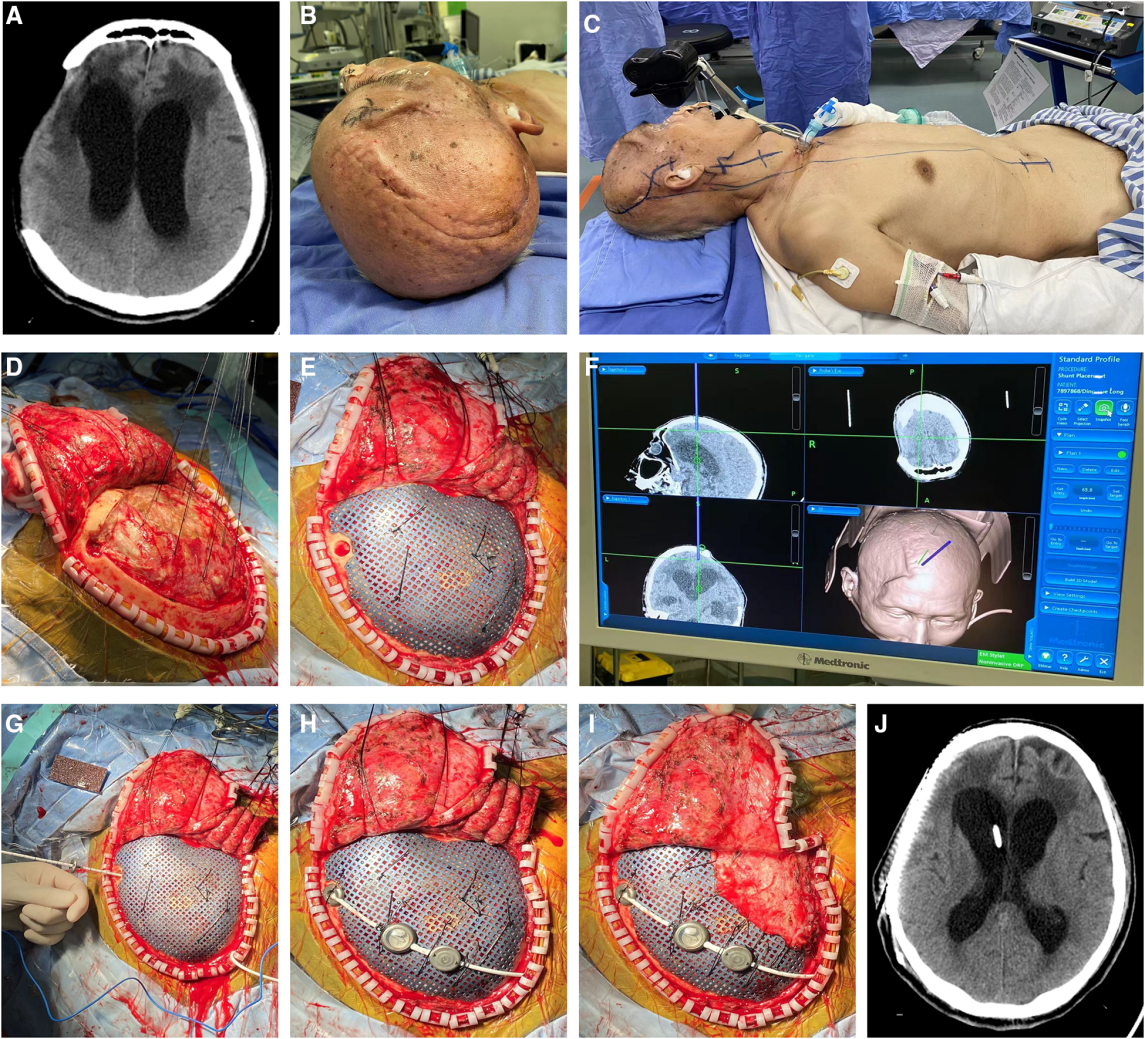
(**A**) Head CT scan showed right cranial defect and dilation of the ventricle system. (**B,C**) According to strategy 1, cranioplasty was performed first. Shunt valve pressure was set at 160 mm H_2_O before the surgery. (**D**) Potential epidural space disappeared after suspending with silk thread between titanium mesh and dura mater. (**E**) The entry point was located by the neuronavigation system on the right frontal bone. (**F**–**I**) Then, ventricular puncture was implemented and the shunting tube was connected. (**J**) No subdural/epidural fluid collection or hemorrhage was revealed by postoperative head CT.

A 74-year-old man was transferred to the local hospital because of a traffic accident. Prompt head CT demonstrated right frontotemporal contusion and acute subdural hematoma. Based on the symptom and examination, the patient received emergent craniectomy. Due to lowering of consciousness, the patient came to our hospital 4 months later from the rehabilitation hospital. Head CT scan showed right cranial defect and dilation of the ventricle system (Figure 6A). We then performed one-stage cranioplasty and VPS. According to strategy 1, we performed cranioplasty first (Figures 6B–E). Shunt valve pressure was set at 160 mm H_2_O before the surgery. Potential epidural space disappeared after suspending the titanium mesh and dura mater with silk thread. The entry point was located by the neuronavigation system in the dura matter and titanium mesh (Figures 6F,G). Then, ventricular puncture was implemented and the shunting parts were connected (Figures 6H,I). No subdural/epidural fluid collection or hemorrhage was revealed by postoperative head CT (Figure 6J).

## Discussion

Decompressive craniectomy is a life-saving method to reduce ICP effectively in some deadly intracranial hypertension. The “syndrome of the trephined” and hydrocephalus are the most common complications of decompressive craniectomy after head trauma, which cause neurologic symptoms (14). Previous studies demonstrated that 11.9%–36% of the patients after decompressive craniectomy progressed to hydrocephalus. Some literature studies reported that traumatic hydrocephalus after DC was closely associated with poor prognosis (15–17). Scholars showed that the cranial box was changed from a closed system to an open one after decompressive craniectomy, thus leading to the increase of brain compliance and reduction of ICP (18, 19). After 1 month, post-traumatic hydrocephalus may occur due to the alterations in dynamics of cerebral blood flow and cerebral spinal fluid caused by the open box system (17, 20, 21). The potential mechanisms were suggested by some literature studies, including arachnoid adhesions resulting in the disruption of CSF circulation, disruption of CSF dynamics, and damaged venous drainage to the sagittal sinus (18, 19, 21, 22).

If hydrocephalus develops in the presence of skull defect, the patient's recovery process will become more complex and unpredictable (3, 23, 24), which may cause neuronal loss and severe atrophy of brain parenchyma, blood blocking, and imbalance of the atmospheric pressure and cerebral perfusion pressure, and lead to the inward displacement of the scalp and decrease of CSF flow over the convexity (25). If hydrocephalus develops after DC in a patient suffering from head trauma, cranioplasty and VP shunting may be necessary. Titanium mesh is a widely used material in cranioplasty, which has good histocompatibility, cosmetic effect, and low cost (26). Polyetheretherketone (PEEK) has been gradually applied in the cranioplasty reconstruction in recent years because of many advantages compared with other materials, including good histocompatibility, low thermal conductivity, resistance to ionizing radiation, and similar properties with native cortical bone (27). Signorelli et al. (28) shared the first clinical data of transcranial sonography after PEEK cranioplasty. High-quality images of the cerebral and ventricular structure were obtained at the patient's bedside, compared with the CT scan results. The shunting catheter was also observed through the PEEK cranioplasty window by transcranial sonography. Transcranial ultrasound may be a simplified and supplementary method in the assessment of ventricular structure of patients after PEEK cranioplasty and shunting surgery.

The ideal approach for resolving both hydrocephalus and cranial defect remains unclear. Some literature studies have showed advantages of staged procedures of cranioplasty and VPS. Schuss et al. found in their study an overall complication rate of 27% in patients who underwent simultaneous CP and VPS experiencing complications more often than those who underwent staged CP and VPS procedures (47% vs. 12%). They showed that simultaneous CP and VPS had a significantly higher rate of infection compared with patients with staged procedures (29). Heo et al. enrolled 51 patients with a skull defect and hydrocephalus and found a higher frequency and severity of overall complication in the simultaneous procedure group compared to the separate procedure group (56% vs. 21%) (11). Morton et al. revealed that the overall complication rate with simultaneous surgical procedure was as high as 56% (30). Pachatouridis et al. also reported that cranioplasty and ventriculostomy followed by the further placement of a VP shunt are associated with fewer complications in the treatment of hydrocephalus after DC (25).

Other scholars found different results in their studies. Meyer et al. (31) compared the one-stage procedure of cranioplasty and VP shunt with separate operations and showed that cranioplasty- and VPS-related infections occurred in both groups (4/26 in the one-stage operation group VS 4/24 in the separate operation group), with no statistically significant difference in morbidity. Jung et al. introduced a one-stage operation with temporary occlusion of the distal shunt catheter. After 5–7 days, a bedside operation was performed to open the distal end, which effectively decreased the risk of subcutaneous fluid and epidural hematoma (32). The same findings were also shown in the study of Zhou et al. (33). In their study, 54 patients underwent simultaneous VPS and cranioplasty vs. 30 who had the procedures performed separately, with no difference in the overall rate of complication. Carvi et al. found that the complications of intracranial hematoma and subdural hygroma were prevented by temporarily occluding the distal shunt catheter and thus inducing adhesion between the dura mater and cranium in a one-stage operation. The occlusion device could easily be taken by a simple surgery without injuring the wall structure of the shunt catheter (34). Furthermore, they revealed that complications in staged operation were associated with general anesthesia and surgery. In our center, 70 patients were divided into two groups for simultaneous and staged procedures of CP and VPS. The results showed that complications suffered by patients who underwent one-stage procedure of CP and VPS did not differ significantly, compared with patients who underwent staged procedures. In the latest years, we tend to choose simultaneous surgeries of CP and VPS. Reasons are as follows (35): (1) Single VPS can effectively reduce the intracranial pressure, but it is difficult to adjust the valve pressure to a steady status in the absence of cranioplasty, which makes cranial box into a closed system. (2) Advantages to the simultaneous approach include more efficient utilization of medical resources, reduced economic and logistical burden for the family and society, and decreased injury of anesthesia/surgery associated with separated procedures. (3) We tend to perform simultaneous cranioplasty and VPS in the same side of cranioplasty, which leaves space for the second surgery in the other side if the original shunting system was dysfunctional. (4). The preoperative pressure of shunt valve was adjusted to 16 cm H_2_O, and silk thread was used to suspend the titanium mesh and dura mater. The method effectively decreased the risk of overdrainage of CSF and the formation of epidural hematoma. Therefore, our improved one-stage cranioplasty and VPS is a feasible procedure to treat post-traumatic hydrocephalus following decompressive craniectomy.

## Conclusions

Our preliminary results indicate that the improved simultaneous procedure of cranioplasty and VPS can be an effective alternative method to treat cranial defect and post-traumatic hydrocephalus with a relatively low risk of complication and can overcome the disadvantage of the previous one-stage operation.

## Data Availability

The original contributions presented in the study are included in the article/Supplementary Material, further inquiries can be directed to the corresponding author.

## Limitations of this study

Our study may have biased results due to retrospective data analysis from a single center. Larger patient population should be enrolled, and further randomized superiority studies are needed to evaluate the safety and effectiveness of one-stage cranioplasty and VP shunting.
